# A rehabilitation intervention to improve recovery after delirium in older people: Mixed methods process evaluation of the RecoverED multi‐site feasibility study

**DOI:** 10.1002/alz70860_102722

**Published:** 2025-12-23

**Authors:** Shruti Raghuraman, Aseel Mahmoud, Alison Bingham, Abigail Laverick, Kirstie Chandler, Linda Clare, Louise M Allan, Sarah Morgan‐Trimmer

**Affiliations:** ^1^ University of Exeter, Exeter, Devon, United Kingdom; ^2^ University of Exeter, Exeter, United Kingdom; ^3^ NIHR Applied Research Collaboration South West Peninsula, Exeter, United Kingdom; ^4^ University of Exeter Medical School, Exeter, United Kingdom

## Abstract

**Background:**

A process evaluation was conducted alongside a multi‐site feasibility trial of RecoverED, a multicomponent delirium rehabilitation intervention for older people in post‐acute settings. Up to 10 sessions of the home‐based intervention is delivered by a multidisciplinary healthcare team. A modified Conceptual Model for Implementation Fidelity was used. Findings on implementation and acceptability are presented.

**Method:**

A mixed‐methods design was employed, and participants included older adults with delirium, their carers, and trained healthcare professionals (HCPs) from six NHS hospitals in the UK. Adherence to content, dose, and coverage, as well as moderating factors such as recruitment, context, participant responsiveness, delivery quality, and intervention complexity, were assessed. Data included in‐depth interviews, focus groups, trial documentation, and training, and supervision logs. Mixed‐methods findings were triangulated.

**Result:**

Nineteen participant‐carer pairs were recruited. Five older people, 9 carers, and 8 HCPs were interviewed post‐intervention. Seven HCPs participated in two focus groups. Evaluating adherence to content was complex since the intervention is person‐centred and personalized. Psychosocial support was delivered more frequently than planned for each individual, while physical rehabilitation and functional recovery activities were delivered less than planned. The value of participant‐led goals was emphasised, with high levels of satisfaction, engagement, and perceived value. Implementation was according to the theorised delivery approach, and participants expressed positive views on the quality of delivery. While HCPs found the training comprehensive, they preferred a more interactive and practical format. Teams need to be specifically staffed and co‐located for effective coordination and supervision of RecoverED. Most withdrawals (*N* = 10) were due to complex needs or impairments. No minority ethnic participants were recruited.

**Conclusion:**

The RecoverED intervention was found to be acceptable; however, recruitment challenges suggest that acceptability and fidelity to dose and coverage should be interpreted with caution. Implementation fidelity to the delivery approach was high and well‐perceived.

